# Essential thrombocythemia with (type2) calreticulin presented as stuttering priapism case report and review of literature

**DOI:** 10.1002/ccr3.3541

**Published:** 2020-11-18

**Authors:** Elrazi Awadelkarim Ali, Abdulqadir Jeprel Nashwan, Mohamed A Yassin

**Affiliations:** ^1^ Internal Medicine Department Hamad Medical Corporation Doha Qatar; ^2^ Nursing Department Hamad Medical Corporation Doha Qatar; ^3^ Hematology and Oncology Department Hamad Medical Corporation Doha Qatar

**Keywords:** calreticulin, erectile dysfunction, essential thrombocythemia, male fertility, myeloproliferative neoplasms, priapism

## Abstract

Priapism is a rare presentation and complication of ET that might be underreported. In ET, priapism can present as an ischemic or stuttering type. These patients are more likely to be anemic and have a high platelet count.

## Graphical abstract

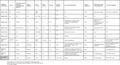

## 1 BACKGROUND

Essential thrombocythemia is a myeloproliferative neoplasm (MPN), characterized by clonal proliferation of myeloid cell line with a significant increase in platelet count. Diagnosis is made by meeting the 2016 WHO criteria with four major or three major and one minor criterion after excluding secondary causes of thrombocytosis. It usually an asymptomatic disease or presents with hemorrhagic or thrombotic complications. Priapism is a prolonged penile erection not related to sexual stimulation. The longer the duration of priapism, the higher the risk for penile fibrosis and erectile dysfunction, which have a devastating outcome on male fertility. Priapism is seen more commonly in hematological conditions, that is, sickle cell anemia. In myeloproliferative neoplasms, it is relatively more common with chronic myeloid leukemia and is rarely reported with essential thrombocythemia, polycythemia Vera, or primary myelofibrosis. We are reporting a case of a 51‐year‐old man with persistent thrombocytosis diagnosed as essential thrombocythemia with *CALR* (Type 2). The patient developed priapism with the onset of rising in platelets count. This is the first report in the literature of *CALR* type 2 essential thrombocythemia presenting with priapism. The case sheds light on rare or underreported complication and reviewing our current knowledge concerning such rare complication.

Priapism is a persistent erection of the penis that is not associated with sexual stimulation or desire lasting more than 4 hours.^1^ Priapism can be classified as ischemic, nonischemic, or stuttering. Stuttering priapism is defined; recurrent priapism lasting less than 4 hours.^2^ Commonly, no cause is identified; secondary causes include medications, especially intracorporal injections or medical conditions such as hematological diseases, particularly sickle cell disease. But it is uncommon to be seen with essential thrombocythemia (ET). It adversely affects the quality of life, sexual function (SF), and physical wellness of the affected patients,^3^ besides the risk of penile fibrosis and permanent erectile dysfunction.^2^, ^4^ The underlying pathophysiology is not clearly understood; several mechanisms have been postulated. This includes disturbed nitrous oxide (NO), phosphodiesterase enzyme activity, and disturbed autoregulation of the penile circulation.^1^, ^5^ There are few reported cases of priapism as the presenting feature of ET.

## 2 CASE REPORT

**FIGURE 1:**
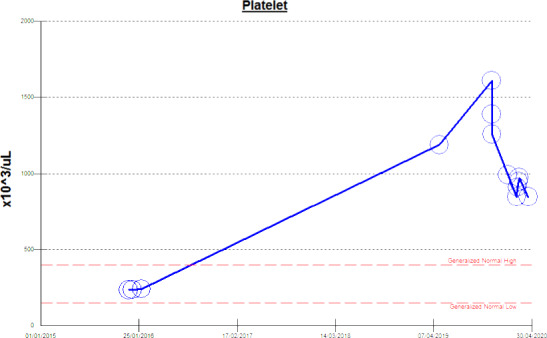
Shows previously normal platelet count until the time of presentation on April 30, 2019

A 51‐year‐old gentleman with a past medical history of sleeve gastrectomy in 2014 and gastric bypass 2016 for morbid obesity, married with no children and referred from a primary healthcare center for evaluation of high platelets, discovered incidentally during a medical checkup. During that time, he reported recurrent painful erections two to three times per month, lasting 15‐60 minutes. No history of trauma or medication use. He has no headache, dizziness, tinnitus, chest pain, or bleeding from any site. No family history of hematological or connective tissue diseases. He had previous normal platelet count with range of (200‐260 × 10^3^/μL) until age of 50 years, then found to have platelet count 1189 × 10^3^/μL (150‐400 × 10^3^/μL) (Figure 1), had hemoglobin level of 11.2 gm/dL (13‐17 gm/dL) WBC 8.7 × 10^3^/μ (4‐10 × 10^3^/μ), iron level 7.00 μmol/L (11.6‐31.30 μmol/L) Fe saturation 11% (15%‐45%), transferrin 2.60 gm/L (2‐3.6 gm/L), and ferritin 3.8 mcg/L (24‐336 mcg/L). Anemia was corrected with iron replacement therapy and repeated follow‐up ferritin improved to 529.0 μg/L. ANA and ANCA were negative. Further workup showed BCR/ABL1 t(9;22)(q34;q11.2) negative. Molecular study for *JAK*2 mutation was negative but positive for Type 2 calreticulin mutation. Bone marrow biopsy findings are consistent with a myeloproliferative neoplasm (consistent with ET).

The peripheral blood smear showed increased numbers of platelets with some large and few giant forms. Chromosomal analysis revealed normal Karyotype: 46,XY. US Abdomen showed normal liver and spleen, no ascites, and no organomegaly. After the diagnosis of ET, the patient was counseled regarding the recurrence of priapism. He was started on aspirin 75 mg daily and is following with urology for priapism. He did not require any urological intervention for priapism.

## 3 DISCUSSION

This patient platelet count was normal up to the time of presentation with priapism, as shown in (Figure 1). His only previous conditions were infertility and sleeve gastrectomy. The majority of patients with essential thrombocythemia are asymptomatic, discovered accidentally by chance during routine blood work or investigation for another reason.^6^ Others present with vasomotor symptoms, thrombotic, or hemorrhagic complications.^7^ Priapism is a rare medical condition incidence in the United States is 0.73 per 100 000 men per year.^8^ It is rare for ET to present as priapism. Our literature search revealed 11 reported cases with ET who developed or presented with priapism.

The most common type of priapism is ischemic (low flow), while nonischemic is much rare and related to trauma.^9^ Stuttering priapism results and can lead to ischemic priapism, and they share similar causes.^9^ It thought that the initial insult results in defective penile circulation due to loss of phosphodiesterase type 5 (PDE5) regulatory function which in turn result in altered NO and cyclic guanosine monophosphate signaling mechanisms.^10^, ^11^ The major hematological cause for ischemic priapism is sickle cell anemia. In ET, the mechanism is probably related to high platelet count and platelets sludging in the corpora cavernosa, similar to RBC sludging in sickle cell disease and WBC sludging in leukemia.^12^ Subsequent stasis and disturbed NO and cyclic guanosine monophosphate signaling. This might be precipitated by increased viscosity due to dehydration or release of free hemoglobin^13^ secondary to hemolysis similar to sickle cell anemia, as many patients had low hemoglobin levels, as seen in (Table 1). The table shows that most of hemoglobin levels are in the low to lower normal limits, but no patient had high hemoglobin levels, opposite to what can be expected that polycythemia might increase the viscosity of the blood and enhance thrombosis. This is supported by observing patients with other myeloproliferative neoplasms who developed priapism. In one study 430 patients with chronic myeloid leukemia, those who developed priapism at diagnosis (8 patients) had lower hemoglobin levels compared to chronic myeloid leukemia patients who did not develop priapism.^14^ In the same study, the mean hemoglobin level in patients with priapism was 8.4 ± 2.3 g/dL, compared to a hemoglobin level of 10.8 ± 2.5 g/dL in chronic myeloid leukemia patient's control who did not develop priapism. The age and platelet count did not have a significant effect on priapism in these patients with chronic myeloid leukemia. To the best of our knowledge, there are no reports of cases of adults with polycythemia Vera who devolved priapism, which might indicate that anemia has a sort of synergistic effect with leukocytosis in the pathogenesis of priapism in CML patients. And it might affect the type of priapism as most CML type is ischemic. But it is difficult to generalize this observation to ET. The effect of hemoglobin level on essential thrombocythemia with priapism is not clear, and it is not clear if the hemoglobin level itself is related to the type of priapism due to incomplete information. However, anemia can enhance thrombosis and the risk of thromboembolism.^15^ This effect is not related to reactive thrombocytosis and might be linked to an increase in plasminogen inhibitors or the levels of clotting factors.^16^, ^17^ In the table, the WBC count is variable but at a high level for most patients. From (Table 1), it is noted that some patients presented after years from the onset of priapism. This may indicate the complaint of priapism is underreported, especially for patients with stuttering priapism who develop erection for a shorter duration of time and subsides spontaneously as with our case. While patients with painful ischemic priapism have prolonged, painful erection usually seeks medical attention from the first time. This can be the reason that chronic myeloid leukemia reports of priapism are relatively more common 1.7% compared to ET ^18^ because CML type is predominantly ischemic. At the same time, ET can be ischemic or stuttering.

**TABLE 1:** List of published cases of priapism (listed chronologically)

Published case reports	Age (Y)	Time from onset of priapism to presentation and diagnosis	Type of priapism	PLT ×10^9^/L	WBC ×10^9^/L	Hb g/dL	Type of mutation	Intervention/medication	Year of publication	Splenomegaly below costal margin (cm)	Criteria for diagnosis
Patient 1^32^	60	5 mo	N/A	1500‐3900	12	4.7	N/A	N/A	1960	Hepatosplenomegaly	Bone marrow examination
Patient 2^32^	66	N/A	N/A	700‐4100	2.9‐41	6	N/A	N/A	1960	N/A	N/A
Patient 3^33^	25	36 h	N/A	3000	14	N/A	N/A	Plateletpheresis, aspiration winter procedure then plateletpheresis,/busulfan	1979	Splenectomy due to splenic vein thrombosis 1976,was on anticoagulation until 1977	N/A
Patient 4^12^	34	Short‐term onset	N/A	1245	15.4	13.2	N/A	Blood clot was evacuated from the corpora cavernosa	1981	Splenectomy for ITP	N/A
Patient 5^34^	45	N/A	N/A	1200‐1600	N/A	N/A	N/A	Bilateral cavern‐ostomy	1990	5 cm	N/A
Patient 6^35^	7	N/A	N/A	4370	19	12	N/A	Busulfan	1997	2 cm	N/A
Patient 7‐8^25^	Mean age 12	N/A	N/A	2300‐2900	23.4‐30.6	N/A	N/A	Aspirin/dipyridamole	2000	N/A	The Polycythemia Vera Study Group criteria
Patient 9 ^27^	21	2 y	Stuttering	830	17	12.2	Jak2	Repeated aspiration and instillation with phenylephrine/aspirin 75 mg hydroxyurea	2017	Mild splenomegaly on ultrasound	The revised WHO criteria for PSV, ET, PMF: an alternative proposal. Blood 2008
Patient 10^26^	71	3 d	Ischemic	945	N/A	N/A	Jack2	Aspiration of corpus cavernosum and injection of phenylephrine/hydroxyurea	2019	N/A	2016 WHO criteria
Patient 11^36^	31	24 h	Stuttering	1424	18.3	13	Jack2	Aspiration of corpus cavernosum and injection of phenylephrine/ hydroxyurea acetyl salicylic acid	2020	No splenomegaly	2016 WHO criteria
Our patient 12	51	1 y	Stuttering	1189	8.7	11.2	*CALR* type 2	Aspirin	2020	No splenomegaly	2016 WHO criteria

Note

The table shows the reported cases of ET arranged by chronological order. Before 2008, there was no genetic testing for diagnosis of ET (2008 WHO criteria).

Abbreviation: N/A, No data available.

Penile tissue composes of two spongy erectile tissue called corpora cavernosa and another spongy tissue in the ventral side of the penis called the corpus spongiosum. Priapism usually involves the cavernosal tissue; less commonly, it can affect the spongiosum and glans penis.^19^, ^20^ After 4‐6 hours of erection, tissue damage can be detected at a microscopic level.^21^ And with more prolonged erection, there is a high risk of permanent damage and fibrosis. If it persists more than 24 hours, the risk of permanent erectile dysfunction is more than 90%^22^ and a drastic effect on male fertility.

Most of these cases are reported a long time ago, and few are reported after the 2008 WHO criteria for ET, which explains why no genetic studies were done for the cases before 2008. Two cases were *Jak2* +, and our case is the first case with *CALR* type 2 ET to present with priapism. Usually, the platelet count is higher with *CALR* mutation than *Jak2* mutation^23^ but with fewer thrombotic or hemorrhagic complications than *Jak2*. Due to the limited numbers of reported cases, it is not clear if the priapism is related to *Jak 2* mutation over *CALR* mutation. As *Jak2* mutation is more common, it is expected to be seen more with *Jak2* than *CALR* mutation. Other ET patients who developed priapism also had high platelet count (830‐4100) (Table 1). Some patients had splenomegaly. Splenomegaly is seen in up to 51% of ET patients by using ultrasonography.^24^ In table, 6/11 had splenomegaly or splenectomy. This ratio is close to ET patients without priapism. The effect of age is ill‐defined, as it was reported in children ^25^ as well in a patient aged 71 years^26^; it seems that age plays a minor role, if any. Additionally, priapism pathophysiology is not clear, and ET presented with different types of priapism stuttering and ischemic,^26^, ^27^ which makes the underlying mechanism of such priapism more debatable.

Generally, stuttering priapism management depends on the situation at presentation. If the patient is in acute priapic even, priapism will be managed as Ischemic priapism.^9^ If the patient is not in a priapic state, then the aim is to prevent the recurrence of the condition.^28^ Management starts with patient counseling regarding early measures to control priapism‐like physical activity, urination, ejaculation, fluid intake, and cold baths.^29^ Various medications had shown benefits. Intracavernosal injection of α‐agonists like phenylephrine or oral‐like pseudoephedrine is used to contract the smooth muscles allowing detumescence.^30^ Other modalities include the use of hormonal therapy to suppress testosterone‐like goserelin acetate, leuprolide, and ketoconazole. Additional effective medications include phosphodiesterase 5 inhibitors, gabapentin,^31^ and terbinafine.^30^

## 4 CONCLUSION

Priapism is a rare presentation and complication of ET. It can present as ischemic or stuttering types. Stuttering priapism might be underreported which explains the fewer reports in ET compared to CML because in CML priapism is mostly low flow type. The main risk factors are not well‐understood due to the old and few reported cases. Splenomegaly, age, and type of mutation appear to have little impact. However, the distinctive feature is the high platelet count with or without anemia but no polycythemia. Early diagnosis and rapid management of such cases could lead to favorable prognosis and better outcome preserving the erectile ability of corpora cavernosa.

## CONFLICT OF INTEREST

None declared.

## AUTHOR CONTRIBUTIONS

EAA: concept, writing, editing and approval of final manuscript. MAY: concept, writing, editing and approval of final manuscript. AJN: writing, editing, and approval of final manuscript.

## CONSENT

The consent for publication was obtained.

## Data Availability

All data generated during this published case report are not publicly available. However, it is available up on request from the corresponding author with permission.
